# The liberating dimension of human habit in addiction context

**DOI:** 10.3389/fnhum.2014.00664

**Published:** 2014-08-28

**Authors:** Francisco Güell, Luis Núnez

**Affiliations:** ^1^Mind–Brain Group, Institute for Culture and Society, Universidad de NavarraPamplona, Spain; ^2^Centro Médico PamplonaPamplona, Spain

**Keywords:** habit, motivation, stimulus-response, addiction, addict behavior, goal-directed behavior, neuropsychological impairment, liberating dimension

The notion of habit has acquired an important role within studies of drug addiction and dependence. In general, classical models of addiction conceive of learned compulsive behaviors in terms of a unidirectional stimulus-response model, for which habits are behavior patterns based on studies of animals and are considered to be purely automated—that is, inflexible, highly stimulus bound and insensitive to associated outcomes (Tiffany, 1990; Miles et al., 2003; Everitt and Robbins, 2005). For this approach, learning converts behavior into an automatism, or what some have termed an addictive habit (for example, Hogarth et al., 2013; Sjoerds et al., 2013). Some of these models have been expanded to incorporate motivational aspects of addiction. Such models regard reinforcement (positive or negative) as the initial and central drive for drug abuse (Robinson and Berridge, 1993; Baker et al., 2004) and are situated in a context of a larger, goal-directed, decision-making framework (Cox and Klinger, 1988; Siegel, 2005; Wes, 2006).

Within this overall picture, Sjoerds's team has proposed to expand the habit formation model by distinguishing between motor habits and motivational habits (Sjoerds et al., 2014). In the case of motor habits, behavior is based on a stimulus-response model, while motivational habits refer to compulsive behavior that is controlled by an emotional/motivational state and seems to be at least partially goal-directed. Sjoerds's proposal is a marked improvement over a strictly motor-habit notion of addiction, but we believe that it still falls short of the full context in which the notion of habit acquires its full significance. Let us examine this context more closely.

To be sure, all existing theoretical models have contributed to the understanding of drug consumption, abuse, and addiction. Generally, they affirm that habitual addictive behaviors are related to reinforcement and are conditioned by the presence of diverse environmental and motivational factors associated with the moment of consumption. With continuous consumption, the subject gradually consolidates a behavior associated with the results of consuming and, with time (a period which some designate as the appearance of substance dependency; Peer et al., 2013), the behavior becomes more and more compulsive and less flexible. Studies point out that the routine behavior responsible for addiction leads to the appearance of a state of allostasis wherein individuals take drugs no longer to feel “high,” but just to feel “right” (Koob and Le Moal, 1997, 2001, 2005; Piazza and Deroche-Gamonet, 2013).

We find it interesting to note how habits are understood within this context. In studies of addiction, “habits” typically refer only to acquired behaviors that incite the subject to consume. That is, regardless of their flexibility and their relation to motivational states, habits acquired by drug addicts are considered to be those specific pathological behaviors that must be eliminated or counteracted. However, within a therapeutic framework, we find a much richer picture of habit.

Basically, such therapies pursue a modification of all the behaviors that are responsible for the consumption of drugs. The principal objective of many approaches is to fight addiction by means of learned techniques for avoiding stimuli associated with the substance (e.g., substance availability, conditioning social and living places, social groups, etc.; Tucker et al., 1990-1991). The problem is that techniques that focus on the elimination of addictive habits do not reinforce the essential supports for what is referred to as personal re-education. In the therapeutic-educative context, it is evident that one of the central consequences of addiction is the loss of habits that are necessary for personal and social realization and which are normally acquired over the course of a healthy life. Of course, if the only objective of the consumer is to obtain the substance so as to avoid the symptoms of withdrawal, any routine behavior that does not have this objective will be useless. But the problem—and here is the crux of the question—is not that addicts have lost or forgotten their daily routines. From a neuropsychological perspective (Robinson and Berridge, 2003; Verdejo-García and Bechara, 2009; García et al., 2011), it has been shown that continuous consumption of drugs deteriorates certain executive functions such that, even after abstinence has begun, cognitive flexibility in the motorization of strategies is reduced and, as a result, the capacity to organize, plan and supervise one's own daily behavior is diminished (Verdejo-García et al., 2004; Verdejo-García, 2005). The addict cannot effectively confront his addiction until this capacity is restored.

Because in-treatment therapeutic communities (such as “Proyecto Hombre”) provide a controlled environment that helps addicts to “kick the habit” and offers treatment for drug abuse, they are an ideal context for scientific research (for example, Verdejo-García, 2007). A quick look at these communities shows that one of the principal problems of drug addicts during the initial and voluntary rehabilitation process is the difficulty in acquiring daily basic routines (Daley, 1989; Verdejo-García, 2005; García et al., 2011). In the scientific literature, there are studies that suggest that rapid recovery of cognitive function during abstinence seems possible (Bates et al., 2005; Rapeli et al., 2006; Schrimsher and Parker, 2008). Anyway, in those therapeutic communities it is well know that It takes months before drug addicts are able to have a natural and reasonable daily routine and to freely assume everyday life activities—and this is only the basis for confronting addiction.

Accordingly, the following paradox arises: while most models of addiction tend to consider habits only as pathological behaviors that push the patient toward continued consumption, many therapies aim precisely at recovering *the capacity to acquire habits*, which has been damaged by continued drug use. In short, from the addiction standpoint, habit is something that needs to be eliminated, and from a therapeutic point of view, it is something that needs to be re-established. In reality, treatment consists in a mixture of both elimination and reestablishment, and both elements are considered to be of equal importance in addiction treatment for the addict, his family and his friends (Thurgood et al., 2014).

In light of this wider therapeutic context, it is evident that habits encompass much more than what is normally defined as the “habit” of drug addiction. Granted, to group all relevant behaviors under the rubric of habit does not correspond with the rigor usually demanded by science. Also, we recognize that scientific understanding of the biological basis of addiction has been advanced by simplified stimulus-response models based on tests with animals, and that the current use of terms like habit, grounded in this experimental context, has proved useful up to a point. However, as has been shown here, it is the actual scientific community that is beginning to notice the limitations of this notion of habit within more complex contexts relevant to human behavior.

Moreover, while the motivational dimension proposed by Sjoerds constitutes a significant improvement over the notion of habit as merely stimulus-response conditioning, this expanded notion of habit could still be interpreted as merely a conditioned response to a stimulus that incorporates a motivational dimension. Our suggestion is that only by taking into account the fuller, the liberating dimension of habit that is revealed in the therapeutic context can we break free from the stimulus-response model. We believe that this liberating dimension, which regulate the disposition of the subject to facilitate certain daily routines and thereby enable the subject to take on other tasks (Güell, 2014), should be acknowledged in the study of drug dependencies as the characteristic and distinctive dimension of human habits.

## Conflict of interest statement

The authors declare that the research was conducted in the absence of any commercial or financial relationships that could be construed as a potential conflict of interest.
